# Association of Childhood Acute Leukemia With Autoimmune Diseases

**DOI:** 10.1002/ijc.70491

**Published:** 2026-04-14

**Authors:** Ida Pellikka, Julia Ventelä, Anssi Auvinen, Olli Lohi, Atte Nikkilä

**Affiliations:** ^1^ Faculty of Medicine and Health Technology Tampere University Tampere Finland; ^2^ Faculty of Social Sciences Tampere University and Tampere University Hospital Tampere Finland; ^3^ Tampere Center for Child, Adolescent, Maternal Health Research and Tays Cancer Center Tampere University and Tampere University Hospital Tampere Finland

**Keywords:** acute leukemia, autoimmune diseases, epidemiology, pediatrics

## Abstract

Emerging evidence indicates a possible association between acute leukemia and type 1 diabetes mellitus (T1DM). However, the relationship between acute leukemia and other autoimmune diseases (AIDs) remains less well understood. To address this gap, we conducted a case–control study using detailed Finnish register‐based data. We identified 1626 childhood leukemia cases (diagnosed 1990–2019) from the Finnish Cancer Registry and sampled three age‐ and sex‐matched controls per case. Information on 29 AIDs, based on previous literature, was obtained from the Care Register of the Finnish Institute for Health and Welfare and on T1DM from the Social Insurance Institution of Finland. Subjects with Down syndrome were excluded. Conditional logistic regression, adjusted for maternal smoking and large for gestational age, was used to estimate associations. After excluding T1DM, AIDs remained associated with an increased risk of childhood leukemia (OR 1.8, 95% CI 1.4–2.4), the association primarily driven by acute lymphoblastic leukemia (OR 1.9, 95% CI 1.4–2.6). Of the AIDs, the association was strongest with rheumatoid diseases (OR 2.6, 95% CI 1.3–4.9). Using lag periods to account for potential sources of bias did not materially change the results. Childhood leukemia, particularly acute lymphoblastic leukemia, was associated with a higher prevalence of AID, independent of T1DM. The findings warrant further investigation on potential shared immune dysregulation or environmental triggers.

## Abstract (highlights)

What's new?

A possible link exists between various pediatric autoimmune diseases (AIDs) and increased risk of childhood leukemia. Although immune dysregulation is a key feature of these conditions, the connection between them remains unclear. In this study, using cancer registry data in Finland, the authors examined associations between AIDs and childhood acute leukemia. Increased leukemia risk, particularly acute lymphoblastic leukemia (ALL) among infants diagnosed within the first year of life, was connected to AID occurrence. The strongest associations were observed with rheumatoid diseases. The findings highlight the need for further research into shared biological mechanisms between childhood AIDs and leukemia.

## Graphical abstract

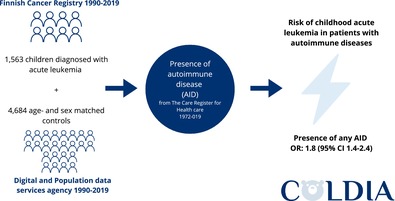

**Abbreviations**
AID: autoimmune disease
ALL: acute lymphoblastic leukemia
AML: acute myeloid leukemia
CI: confidence interval
ICD‐O‐3: International Classification of Diseases for Oncology 3rd Edition
ITP: immune thrombocytopenia
JIA: juvenile idiopathic arthritis
LGA: large for gestational age
OR: odds ratio
RA: rheumatoid arthritis
SIR: standardized incidence ratio
T1DM: type 1 diabetes mellitus
UC: ulcerative colitis

## 1 Background

Childhood acute leukemia is the most common pediatric cancer worldwide [1] with acute lymphoblastic leukemia (ALL) being the predominant subtype [2, 3].

The etiology of ALL is multifactorial, involving a complex interplay between genetic predisposition (somatic and germ line), environmental exposures and immune dysregulation. Leukemia susceptibility often originates from prenatal genetic mutations, but these early lesions are insufficient on their own as additional postnatal genetic alterations are required for full leukemogenesis to develop [4, 5, 6, 7]. Genomic studies have identified germline genetic variants such as *IKZF1*, *CDKN2A*, and mutations in genes like *ETV6*, *TP53*, and *PAX5* as increasing the risk of ALL [8, 9, 10, 11]. Children with certain congenital syndromes such as Down syndrome exhibit markedly increased risk of developing acute leukemia [12, 13, 14].

Factors that drive secondary changes leading to onset of leukemia are not fully understood [7]. An abnormal immune response to infections during childhood has been considered a major contributing factor, although its precise underlying mechanisms remain unclear [4, 6, 7, 15].

An increasing body of evidence suggests potential links between acute leukemia and other non‐communicable diseases, particularly autoimmune diseases (AIDs) [16, 17, 18, 19]. AIDs are a diverse group of diseases where the body's immune system falsely attacks its own tissues [20]. The most prevalent childhood AIDs include type 1 diabetes mellitus (T1DM), celiac disease, juvenile idiopathic arthritis (JIA), and inflammatory bowel disease [21, 22, 23]. Childhood acute leukemia and AIDs have multifactorial etiologies, involving genetic predisposition and environmental factors. Notably, they share a common feature: dysregulation of the immune system [24, 25]. In addition, autoimmune phenomena may develop after leukemia treatment, potentially related to immune reconstitution, therapy‐induced immune dysregulation, or late effects of treatment [26, 27].

Our prior systematic review examined links between childhood acute leukemia and AIDs, showing a 2.2‐fold increased leukemia risk [28]. In a related study [29], we demonstrated a twofold increased risk linking T1DM and leukemia. Here, we aimed to investigate the risk of acute leukemia among pediatric patients diagnosed with AIDs other than T1DM in a nationwide register‐based case–control study. Additionally, we sought to determine whether this association varied by type of leukemia or AID.

## 2 Materials and Methods

### 2.1 Data

Childhood acute leukemia cases were identified from the Finnish Cancer Registry using the International Classification for Diseases of Oncology, 3rd Edition (ICD‐O‐3), specifically morphology codes M9800–M9948 [30]. We included acute leukemia cases diagnosed in individuals under 18 years of age between 1990 and 2019. The cases were further divided into ALL and acute myeloid leukemia (AML) based on ICD‐10 and ICD‐O‐3 morphology classifications.

For each case, three sex‐ and age‐matched controls were randomly selected from the Finnish Population Information System. Each control was assigned a reference date corresponding to the age at which their matched case was diagnosed.

Information on AIDs for the study population was retrieved from national health care registers. Data from 1972 to 2019 was obtained from the Care Register for Health care, and for earlier years (1972–1993), from the Hospital Discharge Register. The AID was collected if it was marked as the primary or the secondary diagnosis of the visit. A total of 29 AIDs were included, based on the study [25], and identified using ICD‐9 and ICD‐10 codes (Table S1). Additionally, we collected the date of first health care contact with the diagnosis to estimate the diagnosis date of each disease. No age restrictions were applied to AID diagnoses, which could occur either before or after the leukemia diagnosis. T1DM diagnoses were identified using reimbursement data from the Social Insurance Institution of Finland [29].

To account for potential confounding factors, demographic data were supplemented with information on Down syndrome and birth‐related variables including large for gestational age (LGA) and maternal smoking status from the Medical Birth registry. As described in our previous study, planned caesarean section and maternal age showed no significant associations with leukemia risk and were therefore excluded from the multivariate model to preserve statistical power [29]. Data on Down syndrome were obtained from the Register of Congenital Malformations, and leukemia cases and controls diagnosed with Down syndrome were excluded from the analyses (63 cases and 4 controls).

### 2.2 Statistical Analysis

The associations between childhood acute leukemia and AIDs were evaluated with conditional logistic regression using the *survival* package in R. We first created a univariate model with AID as an independent variable to calculate crude odds ratios (ORs) and 95% confidence intervals (CIs) for leukemia. Additionally, we assessed leukemia risk according to the temporal order of the diagnoses, distinguishing between leukemia diagnosed before AIDs and leukemia diagnosed after AID. Subsequently, we adjusted the model with potential confounders using multivariable logistic regression.

We first performed analyses examining AIDs excluding T1DM (Table S1), which were then followed by analyses stratified by groups of AIDs and by individual conditions. To assess the robustness of our findings and potential misclassification bias, we conducted a series of sensitivity analyses with varying criteria based on the timing of AID relative to leukemia. These criteria were defined by comparing the dates of AID and leukemia (Figures S1 and S2) to ensure that leukemia‐related symptoms were not misclassified as AIDs. Suspicious diagnoses in the Care Register were manually reviewed at the patient level, examining individual patient records. Based on this review, lag times were determined, and certain diagnoses were excluded from the analyses.

We applied reclassification of AID exposure status according to predefined timing criteria. Specifically, AID was reclassified for diagnoses occurring within 90 days prior to or within 1 or 2.5 years after leukemia diagnosis including combined intervals. At the condition level, AID was reclassified for idiopathic thrombocytopenic purpura (ITP), Crohn's disease, or ulcerative colitis (UC) within 30 days, and Addison's disease or juvenile arthritis within 60 days of leukemia diagnosis and rheumatoid arthritis (RA) 90 days prior to leukemia diagnosis, as these conditions could overlap with early leukemia symptoms or be influenced by treatments initiated around diagnosis.

To further assess the potential contribution of T1DM, we subsequently conducted pooled analyses, in which T1DM was included together with all other AID, with additional exclusion of individuals diagnosed with pancreatitis to minimize potential confounding.

Statistical analyses were performed using R (version 4.0.5, R Core Team, 2021, Vienna). All analyses took place within a secure data environment (Kapseli) managed by Findata to ensure patient data confidentiality. A significance level of *p* < 0.05 was applied for all analyses and all *p*‐values were calculated as two‐sided.

## 3 Results

In total, 1765 cases of childhood leukemia were identified from the Finnish Cancer Registry. Cases diagnosed with chronic leukemia or lymphoma were excluded. For cases with multiple leukemia diagnoses (relapsed or a secondary leukemia), only the first diagnosis was retained in the dataset. The final cohort included 1626 leukemia cases with a slight predominance of male patients (*n* = 876, 53.9%). The median age at the time of diagnosis was 5.0 years (IQR 2.9–10.3) and the majority of cases were diagnosed with ALL (*n* = 1350, 83.0%) (Table 1).

**TABLE 1:** Characteristics of the study subjects.

	Cases (%)	Controls (%)
Total, *N* (%)	1626	4877
Sex, *N* (%)		
Female	750 (46.1)	2250 (46.1)
Male	876 (53.9)	2627 (53.9)
Age, *N* (%)		
0–0.99 years	78 (4.8)	234 (4.8)
1–9.99 years	1127 (69.3)	3380 (69.3)
10–17.99 years	421 (25.9)	1263 (25.9)
Type of leukemia, *N* (%)		
ALL	1350 (83.0)	4049 (83.0)
ALL, female	620 (45.9)	1860 (45.9)
ALL, male	730 (54.1)	2189 (54.1)
ALL, 0–0.99 years	47 (3.5)	141 (3.5)
ALL, 1.5–5.99 years	732 (54.2)	2196 (54.2)
ALL, 1–9.99 years	987 (73.1)	2960 (73.1)
ALL, 10–17.99 years	316 (23.4)	948 (23.4)
AML	224 (13.8)	672 (13.8)
AML, female	110 (49.1)	330 (49.1)
AML, male	114 (50.9)	342 (50.9)
Others	52 (3.2)	156 (3.2)
Diagnosed with autoimmune disease		
Any autoimmune disease	121 (7.4)	193 (4.0)
Any autoimmune disease, T1DM excluded	103 (6.3)	162 (3.3)
Type 1 diabetes mellitus	22 (1.4)	34 (0.8)
Addison's disease	8 (0.5)	1 (0.0)
Autoimmune hemolytic anemia	1 (0.1)	0
Autoimmune thyroiditis	4 (0.2)	1 (0.0)
Ankylosing spondylitis	2 (0.1)	9 (0.2)
Grave's disease	4 (0.2)	11 (0.2)
Crohn's disease	6 (0.4)	22 (0.5)
Celiac disease	14 (0.9)	30 (0.6)
Juvenile idiopathic arthritis	11 (0.7)	11 (0.2)
Multiple sclerosis	1 (0.1)	8 (0.2)
Immune thrombocytopenic purpura	13 (0.8)	7 (0.1)
Localized scleroderma	0	1 (0.0)
Pemphigoid	1 (0.1)	3 (0.1)
Pemphigus	2 (0.1)	1 (0.0)
Pernicious anemia	2 (0.1)	1 (0.0)
Polyarteris nodosa	1 (0.1)	1 (0.0)
Dermatomyositis	0	1 (0.0)
Psoriasis	11 (0.7)	30 (0.6)
Rheumatoid arthritis	8 (0.5)	9 (0.2)
Rheumatic fever	4 (0.2)	0
Sarcoidosis	4 (0.2)	2 (0.0)
Sjögren's syndrome	5 (0.3)	0
Systemic sclerosis	1 (0.1)	2 (0.0)
Ulcerative colitis	14 (0.9)	37 (0.8)
Potential confounding factors, *N* (%)		
Pancreatitis	30 (1.8)	2 (0.04)
Down syndrome	63 (3.9)	4 (0.08)
Large for gestational age	68 (5.1)	131 (3.3)
Maternal smoking during pregnancy	225 (17.0)	590 (15.2)

Abbreviations: ALL, acute lymphoblastic leukemia; AML, acute myeloid leukemia.

**FIGURE 1:**
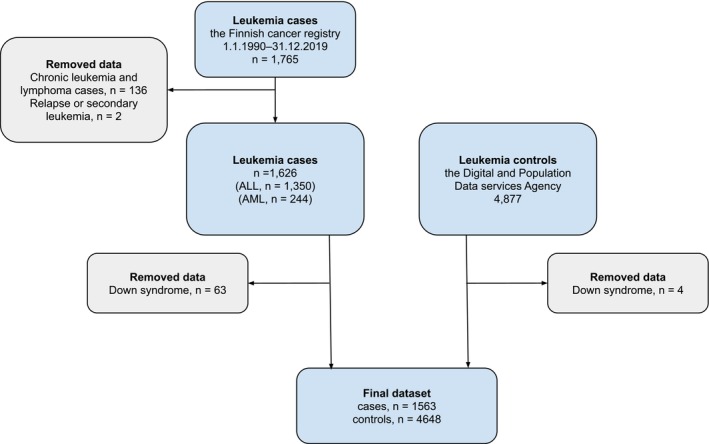
Flowchart of case and control selection for the study. After excluding individuals with Down syndrome (63 cases, 4 controls), the final dataset included 1563 leukemia cases and 4648 controls.

Among the 1626 children diagnosed with acute leukemia, 103 had a diagnosis of AID (55 female, 48 male), with T1DM excluded. The mean interval between AID and leukemia diagnosis was 2080 days; the median was 1270 days, with a range from −3737 to 10,135 days. To reduce potential confounding, all individuals with Down syndrome were excluded. Thereby, the final dataset available for conditional logistic regression comprised 1563 leukemia cases and 4684 matched controls (Figure 1).

### 3.1 Univariate Model

Conditional logistic regression showed that a diagnosis of any AID was associated with an increased risk of acute leukemia (OR 1.84, 95% CI 1.41–2.40) (Table 2). Subgroup analyses indicated the most pronounced association with ALL with the OR of 1.91 (95% CI 1.43–2.55), whereas for AML the OR was 1.20 (95% CI 0.34–2.56). The OR for female patients was 1.78 (95% CI 1.23–2.58) and for males 1.90 (95% CI 1.29–2.79).

**TABLE 2:** Association between childhood acute leukemia and autoimmune diseases.

	Cases exposed	Controls exposed	Univariate model	Multivariate model
	% (*n* =)	% (*n* =)	OR (95% CI)	OR (95% CI)
Any autoimmune disease (T1DM excluded)				
Total (*n* = 1563)	5.9 (92)	3.3 (155)	1.84 (1.41–2.40)	2.05 (1.48–2.84)
Sex				
Female (*n* = 714)	6.7 (48)	3.9 (84)	1.78 (1.23–2.58)	2.01 (1.27–3.20)
Male (*n* = 849)	5.2 (44)	2.8 (71)	1.90 (1.29–2.79)	2.11 (1.34–3.34)
Age				
0–0.99 years old (*n* = 71)	8.5 (6)	0.9 (2)	16.12 (1.92–135.3)	—
1–9.99 years old (*n* = 1081)	5.4 (58)	2.8 (90)	2.01 (1.43–2.83)	1.96 (1.35–2.84)
10–17.99 years old (*n* = 410)	6.8 (28)	5.1 (63)	1.35 (0.86–2.13)	1.57 (0.74–3.33)
Type of leukemia				
ALL (*n* = 1311)	6.1 (80)	3.3 (131)	1.91 (1.43–2.55)	2.04 (1.44–2.89)
ALL, female (*n* = 597)	7.2 (43)	4.1 (73)	1.86 (1.25–2.75)	1.88 (1.15–3.07)
ALL, male (*n* = 714)	5.2 (37)	2.7 (58)	1.97 (1.29–3.01)	2.25 (1.37–3.70)
ALL, 0–0.99 years (*n* = 46)	8.7 (4)	0.7 (1)	—	—
ALL, 1.5–5.99 years (*n* = 710)	5.4 (38)	2.5 (53)	2.28 (1.47–3.52)	2.19 (1.38–3.49)
ALL, 1–9.99 years (*n* = 958)	5.3 (51)	2.9 (83)	1.92 (1.35–2.94)	1.87 (1.25–2.78)
ALL, 10–17.99 years (*n* = 307)	8.1 (51)	5.1 (47)	1.64 (0.99–2.70)	2.19 (0.98–4.86)
AML (*n* = 203)	3.9 (8)	3.3 (20)	1.20 (0.34–2.56)	1.91 (0.68–5.37)

Abbreviations: 95% CI, 95% confidence interval; OR, odds ratio.

In an age‐stratified analysis, the risk of acute leukemia was highest in subjects under 1 year of age (OR 16.12, 95% CI 1.92–135). Among patients who were diagnosed with acute leukemia during infancy, the AIDs observed later in life included Addison's disease (*n* = 1), Crohn's disease (*n* = 2), celiac disease (*n* = 1), JIA (*n* = 2), psoriasis (*n* = 1), ulcerative colitis (*n* = 1), sarcoidosis (*n* = 1), and T1DM (*n* = 1).

Among patients aged 10–17, the OR was 1.35 (95% CI 0.86–2.13). Among ALL patients, the association appeared strongest at the age corresponding to the peak incidence in our dataset (1.5–5.99 years), with an OR of 2.28 (95% CI 1.47–3.01).

We further stratified the data into pre‐school children (0–6 years) and school‐age children (7–17 years). Among all leukemia cases, the estimated OR was 2.44 (95% CI 1.71–3.50) for children aged 0–6 years and 1.30 (95% CI 0.86–1.96) for those aged 7–17 years. A similar pattern was observed for ALL: the OR for children aged 0–6 years was 2.28 (95% CI 1.55–3.35), whereas for those aged 7–17 years the OR was 1.53 (95% CI 0.98–2.37).

To assess the influence of disease chronology, we conducted ancillary analyses incorporating temporal restrictions. A prior diagnosis of an AID was associated with leukemia with an OR of 2.26 (95% CI 1.19–4.26). When considering AID diagnoses occurring after the diagnosis of leukemia, an association of similar magnitude was observed (OR 1.76, 95% CI 1.31–2.36) (Table S2).

Sensitivity analyses incorporating lag times were performed to evaluate potential bias. When AID exposure was excluded for diagnoses occurring within 90 days before or within 1 year after leukemia diagnosis, the association remained (OR 1.38; 95% CI, 1.03–1.86). Using a stricter lag window by removing individuals with an AID diagnosed within 90 days before or within 2.5 years after leukemia resulted in an association that remained essentially unchanged (OR 1.32; 95% CI, 0.97–1.79) (Table S3).

Analyses stratified by AID categories suggested an increased risk of leukemia in the composite group of rheumatic diseases (RA, JIA, or ankylosing spondylitis) with an OR of 2.55 (95% CI 1.34–4.87) (Table S4). For inflammatory bowel diseases (Crohn's disease or ulcerative colitis), the OR was 1.3 (95% CI 0.74–2.13), and for thyroid diseases (autoimmune thyroiditis or Graves' disease), the OR was 1.75 (95% CI 0.69–4.44). Analyses of individual autoimmune conditions were limited by sample size, resulting in imprecise estimates (Table S5).

### 3.2 Multivariate Analysis

In the multivariate conditional logistic regression analysis adjusted for maternal smoking and LGA, subjects with AIDs remained at increased risk of leukemia. The diagnosis of AID was associated with an OR of 2.05 (95% CI 1.48–2.84), with subgroup analyses yielding similar results (Table 2).

### 3.3 Pooled Analyses Including T1DM

To assess the robustness of the primary findings, a pooled analysis that included T1DM with the other AIDs was conducted. To reduce potential confounding, individuals with diagnosis of pancreatitis were excluded (30 cases, 2 controls). The final dataset comprised 1533 cases and 4593 matched controls. The findings were consistent, yielding an OR of 1.80 (95% CI 1.40–2.30) (Table S6).

## 4 Discussion

We found a two‐fold association between AIDs and the risk of childhood acute leukemia. This association remained robust after excluding T1DM and adjusting for central known confounders. Sensitivity analyses incorporating lag times further supported the validity of the results by minimizing the potential influence of reverse causation and diagnostic bias. The association appeared strongest among infants diagnosed with leukemia during the first year of life and across different AID categories, particularly rheumatic diseases. However, the CIs overlapped with those of the smaller effect estimates, and these differences should therefore be interpreted with caution.

Our findings add to a growing body of evidence suggesting a link between AID and leukemia [18, 24, 25, 31]. In a Swedish population‐based study, Hemminki et al. [25] reported an increased standardized incidence ratio (SIR) of 1.44 for leukemia overall, with elevated risk observed across 13 different AIDs. The SIR of any AID for ALL was 1.7, closely aligning with our observed OR of 1.9. Our analysis included a longer list of AIDs which may partly explain why our observed OR (1.9) was slightly higher. Consistent with our findings, the strongest association in their study was seen for rheumatic diseases (SIR 2.8). Similarly, Zhou et al. [18] reported that individuals with AIDs had more than a threefold increased risk of developing leukemia. In that study, a female predominance was noted (female vs. male: SIR 3.6 vs. SIR 2.8) but, in contrast, we did not observe meaningful differences by sex in our analyses. However, as these studies were conducted in adult populations, their results may not be directly comparable to our pediatric cohort.

Previous studies investigating the association between JIA and the risk of lymphoproliferative malignancies, including leukemia, have yielded mixed results. In line with our findings, register‐based cohort studies in Sweden and Taiwan reported increased risk of malignancy among children with JIA [23, 32]. Neither study found evidence that anti‐inflammatory medications, such as methotrexate or biologic disease‐modifying antirheumatic drugs, further increased cancer risk at the population level, suggesting that the observed association could be attributable to the underlying disease or shared etiology rather than treatment. In contrast, a Japanese cohort study reported a decreased risk of leukemia but increased risk of lymphoma in adult patients with RA suggesting that the relationship between chronic inflammatory diseases and leukemia may differ by age group, disease subtype, or underlying pathophysiology [33].

It is also important to consider the potential for diagnostic overlap. Children with acute leukemia may initially present with arthritis and be referred to a rheumatologist, raising the possibility that some early leukemia cases diagnosed as JIA may represent misclassification [34, 35]. To account for this possibility, we applied multiple lag‐time restrictions in our analyses, and the association between AIDs and leukemia remained evident.

The strongest association was observed among cases diagnosed with leukemia during infancy, highlighting the potential role of immune dysregulation in early life. A similar pattern was seen in pre‐school children (0–6 years) with an OR of 2.4 and school‐age children (7–18 years) with an OR of 1.3. Although the CIs overlapped across age groups, the larger point estimates in younger children suggest that the association may be more pronounced in early childhood. This age‐specific pattern aligns with evidence suggesting that prenatal and perinatal immune exposures can shape hematopoietic development and influence cancer susceptibility [4, 5, 6, 7].

Furthermore, the relationship between immune function and leukemia may be bidirectional: survivors of childhood leukemia have been reported to exhibit an increased risk of developing AIDs later in life [26]. Together, these findings support the concept that immune dysregulation and leukemogenesis may share common underlying biological pathways. Alternatively, leukemia treatments administered in early childhood may contribute to subsequent immune‐related problems [27]. This hypothesis is supported by the more consistently elevated point estimates when leukemia was diagnosed before AID, rather than the reverse.

Beyond epidemiological associations, emerging evidence suggests that AIDs and acute leukemia may share common genetic risk factors [36, 37, 38]. In a recently published large‐scale genome‐wide association study, Yu et al. [36] identified 73 loci jointly associated both with B‐ALL and AIDs, including *IKZF1*, *GATA3*, *IKZF3*, *GSDMB*, and *ORMDL3*, implicating pathways related to cytokine signaling, B‐cell activation, and JAK–STAT signaling. These findings highlight that genetic predisposition to immune dysregulation may also increase leukemia risk, reinforcing the biological plausibility of our observed epidemiologic associations.

The principal strength of this study lies in its nationwide, population‐based design and the use of high‐quality Finnish registry data. The Finnish Cancer Registry is a well‐maintained resource with excellent coverage, capturing over 85% of non‐solid tumors [39]. Childhood leukemia care in Finland is centralized in five university hospitals, likely improving data consistency and coverage. The Finnish Care Register for Health care is a nationwide administrative register that collects standardized data on all patients receiving specialized health care in Finland. It covers public and private hospitals, specialized outpatient care, and primary‐care inpatient wards. Reporting is mandatory, and the coverage of the register ranges from satisfactory to very good (positive predictive values from 75% to 99%) [40].

To enhance diagnostic accuracy and minimize misclassification, AID diagnose codes were manually evaluated at the individual patient level. We examined timing between leukemia and AIDs, and cases in which the diagnoses occurred close together were reviewed individually in the care register records. Consequently, multiple sensitivity analyses were performed excluding time periods of different length around the leukemia diagnosis. Pancreatitis cases were excluded from analyses involving T1DM to avoid inclusion of asparaginase‐induced post‐pancreatitic diabetes [41]. Likewise, individuals with Down syndrome were excluded due to their strong predisposition to both leukemia and AIDs [13, 42]. The ability to link data across multiple national registries further strengthens the validity and robustness of our findings.

This study has several limitations, primarily concerning data completeness and diagnostic accuracy. Data from the Medical Birth Register were unavailable for 17% of cases and 19% of controls, largely due to its establishment only in 1987. This disproportionately affected participants born earlier and may have reduced the precision of adjustments for key confounders, mode of delivery, and maternal smoking. As a result, estimates in multivariable models were less precise, although ORs remained consistent. In addition, some potentially relevant exposures, including breastfeeding, early‐life infections, and air pollution, were not available and could not be accounted for, which may have resulted in a small degree of residual confounding.

The relatively small number of leukemia cases with preceding autoimmune diagnoses also limited statistical power, particularly for AML and analyses of specific AIDs. Information on AIDs was derived from the Finnish Care Register for Health Care, which captures nationwide secondary care data but has not been validated to the same extent as the Finnish Cancer Registry. Some degree of diagnostic misclassification is therefore possible, despite efforts to minimize it. Finally, AIDs are heterogeneous, and not all are associated with widespread end organ damage. Some primarily affect a single organ and may be less relevant to pathways leading to leukemia; in this study, we did not investigate these individual AIDs or subtype‐specific associations due to the limited number of cases.

In conclusion, this study provides evidence that children with AIDs have an increased risk of developing acute leukemia, with the association being most pronounced among those diagnosed during early childhood. These findings underscore the potential role of shared immune dysregulation in the pathogenesis of childhood leukemia and AIDs. Future studies with larger cohorts and more detailed clinical data are warranted to validate these observations, delineate the contribution of specific autoimmune conditions, and elucidate the underlying biological mechanisms.

## Author Contributions

**Ida Pellikka:** conceptualization, methodology, software, writing – original draft, data curation, formal analysis, visualization. **Julia Ventelä:** conceptualization, methodology, software, writing – review and editing, data curation, project administration. **Anssi Auvinen:** validation, writing – review and editing, supervision. **Olli Lohi:** conceptualization, methodology, validation, supervision. **Atte Nikkilä:** conceptualization, validation, methodology, supervision, funding acquisition, project administration.

## Funding

This work was supported by Competitive State Research Funding (22036€); Väre Foundation for Pediatric Cancer Research (2800€).

## Ethics Statement

According to Finnish research legislation, ethical approval was not required for this study as it was based on registry data. This study has the research permission from Finnish Social and Health Data Permit Authority (Findata) and the approval number is THL/1096/14.02.00/2022.2.

## Conflicts of Interest

The authors declare no conflicts of interest.

## Supporting information


**Table S1:** List of autoimmune diseases according to diagnosis code.
**Table S2:** Odds ratios for acute leukemia based on the diagnosis order in patients with autoimmune diseases.
**Table S3:** Sensitivity analyses, odds ratios for acute leukemia by latency periods.
**Table S4:** Association between childhood acute leukemia and autoimmune diseases by composite groups.
**Table S5:** Odds ratios for acute leukemia in individual autoimmune diseases.
**Table S6:** Pooled analyses including type 1 diabetes mellitus.
**Figure S1:** Timing of autoimmune disease diagnoses relative to leukemia.
**Figure S2:** Timing of autoimmune disease diagnoses in controls relative to reference date.

## Data Availability

The data that support the findings of this study are derived from Finnish national registries and are accessible after approval of Findata. The source code is publicly available on GitHub (https://github.com/idapellikka/COLDIA_leuk_AID.R). Further information is available from the corresponding author upon request.
