# Supplementary material for: Association of Childhood Acute Leukemia With Autoimmune Diseases
Source: Int J Cancer. 2026 Apr 14;159(5):1188–95. doi: 10.1002/ijc.70491 (PMC13340990; doi:10.1002/ijc.70491)
Supplement: Supplementary file 1 — Table S1: List of autoimmune diseases according to diagnosis code. Table S2: Odds ratios for acute leukemia based on the diagnosis order in patients with autoimmune diseases. Table S3: Sensitivity analyses, odds ratios for acute leukemia by latency periods. Table S4: Association between childhood acute leukemia and autoimmune diseases by composite groups. Table S5: Odds ratios for acute leukemia in individual autoimmune diseases. Table S6: Pooled analyses including type 1 diabetes mellitus. Figure S1: Timing of autoimmune disease diagnoses relative to leukemia. Figure S2: Timing of autoimmune disease diagnoses in controls relative to reference date. [file IJC-159-1188-s001.pdf]

SUPPLEMENTARY MATERIAL

Association of childhood acute leukemia with autoimmune diseases

Ida Pellikka, Julia Ventelä, Anssi Auvinen, Olli Lohi, Atte Nikkilä

Table of contents

Supplementary tables.. 2

Supplementary table S1. List of autoimmune diseases according to diagnosis code........2

Supplementary table S2: Odds ratios for acute leukemia based on the diagnosis order in patients with autoimmune diseases.. 4

Supplementary table S3: Sensitivity analyses, odds ratios for acute leukemia by latency periods..6

Supplementary table S4: Association between childhood acute leukemia and autoimmune diseases by composite groups.. 7

Supplementary table S5: Odds ratios for acute leukemia in individual autoimmune diseases.. 9

Supplementary table S6: Pooled analyses including type 1 diabetes mellitus.................14

Supplementary figures... 15

Supplementary figure S1: Timing of autoimmune disease diagnoses relative to leukemia. 16

Supplementary figure S2: Timing of autoimmune disease diagnoses in controls relative to reference date.. 18

Supplementary tables

Supplementary table S1. List of autoimmune diseases according to diagnosis code.

Autoimmune disease(n=29)	ICD-10-code*	ICD-9-code*
Addison's disease	E27[†]	255.41 / 2554A
Autoimmune hemolytic anemia	D59[†]	283
Autoimmune hepatitis	K75.4	571.42
Autoimmune thyroiditis	E06.3	245.2 / 2452A
Ankylosing spondylitis	M45	720
Basedow's disease	E05.0	242
Behçet's disease	M35.2	136.1 / 1361A
Crohn's disease	K50	555
Celiac disease	K90.0	579.0 / 5790A
Dermatomyositis and polymyositis	M33	710.3, 710.4 / 7103A, 7104A
Granulomatotic polyangiitis	M31.3	446.4 / 4464A
Idiopathic thrombocytopenic purpura	D69.3	287.31 / 2873A
Juvenile rheumatoid arthritis	M08[‡]	714.3 / 7143A-D, 7143X
Localized scleroderma	L94.0	701.0
Multiple sclerosis	G35	340
Pemphigoid	L12	694.5
Pemphigus	L10*	694.4
Pernicious anemia	D51[†]	281.0 / 2810A
Polyarteritis nodosa	M30	446.0 / 4460A-4460C
Polymyalgia rheumatica	M31.5, M35.3	725 / 7250A
Psoriasis	L40	696
Rheumatic fever	I00-I02	390-392
Rheumatoid arthritis	M05-M06	714
Sarcoidosis	D86	135 / 1350A
Sjögren's syndrome	M35.0	710.2 / 7102A, 7102B
Systemic lupus erythematosus	M32[†]	710.0
Systemic sclerosis	M34[†]	710.1 / 7101A
Ulcerative colitis	K51[†]	556

* Primarily selected based on the ICD-10 code. ICD-9 code used only when ICD-10 is unavailable.
† Drug-induced diseases were excluded.
‡ Unspecified juvenile arthritis was excluded.

Supplementary table S2: Odds ratios for acute leukemia based on the diagnosis order in patients with autoimmune diseases

Leukemia diagnosed before autoimmune disease	Cases exposed* % (n=)	Controls exposed* % (n=)	Univariate model OR (95% CI)	Multivariate model OR (95% CI)
Any autoimmune disease (T1DM excl.)				
Total (n=1563)	4.8 (75)	2.8 (132)	1.76 (1.31-2.36)	2.18 (1.51-3.14)
Sex				
Female (n=714)	5.6 (40)	3.4 (72)	1.74 (1.16-2.60)	2.24 (1.33-3.77)
Male (n=849)	4.1 (35)	2.4 (60)	1.79 (1.17-2.75)	2.15 (1.28-3.61)
Age				
0-0.99 years old (n=71)	7.0 (5)	0.9 (2)	13.14 (1.51-114.1)	-
1-9.99 years old (n=1081)	4.3 (47)	2.3 (76)	1.93 (1.33-2.82)	1.99 (1.32-3.00)
10-17.99 years old (n=410)	5.6 (23)	4.4 (54)	1.29 (0.78-2.13)	2.04 (0.82-5.13)
Type of leukemia				
ALL (n=1311)	5.3 (69)	3.0 (119)	1.91 (1.43-2.55)	2.23 (1.52-3.28)
ALL, female (n=597)	6.4 (38)	3.5 (63)	1.91 (1.40-2.75)	2.12 (1.24-3.61)
ALL, male (n=714)	4.3 (31)	2.6 (50)	1.92 (1.21-3.05)	2.41 (1.39-4.18)
ALL, 0–0.99 years (n=46)	6.5 (3)	0.7 (1)	-	-
ALL, 1.5–5.99 years (n=710)	4.6 (33)	2.2 (47)	2.22 (1.40-3.54)	2.21 (1.35-3.62)
ALL, 1–9.99 years (n=958)	4.8 (45)	2.6 (71)	1.99 (1.35-2.94)	2.06 (1.34-3.16)
ALL, 10–17.99 years (n=307)	6.8 (21)	5.3 (41)	1.58 (0.92-2.72)	2.55 (0.97-6.69)
AML (n=203)	3.0 (5)	2.6 (16)	0.94 (0.34-2.56)	2.29 (0.61-8.58)
Leukemia diagnosed after autoimmune disease				
Any autoimmune disease (T1DM excl.)				
Total (n=1563)	1.1 (17)	0.5 (23)	2.26 (1.19-4.26)	1.63 (0.80-3.32)
Sex				
Female (n=714)	1.1 (8)	0.6 (12)	2.06 (0.82-5.15)	1.34 (0.47-3.80)
Male (n=849)	1.1 (9)	0.4 (11)	2.45 (1.02-5.92)	1.96 (0.74-5.22)
Age				
0-0.99 years old (n=71)	1.4 (1)	-	-	-
1-9.99 years old (n=1081)	1.0 (11)	0.4 (14)	2.43 (1.08-5.45)	1.82 (0.75-4.42)
10-17.99 years old (n=410)	1.2 (5)	0.7 (9)	1.67 (0.56-4.97)	0.96 (0.25-3.69)
Type of leukemia				
ALL (n=1311)	0.8 (11)	0.5 (18)	1.86 (0.87-4.00)	1.36 (0.59-3.15)
ALL, female (n=597)	0.8 (5)	0.6 (10)	1.53 (0.51-4.62)	1.04 (0.3-3.65)
ALL, male (n=714)	0.8 (6)	0.4 (8)	2.25 (0.78-6.48)	1.72 (0.55-5.33)
ALL, 0–0.99 years (n=46)	8.7 (4)	0.7 (1)	-	-

ALL, 1.5–5.99 years (n=710)	1.0 (5)	0.3 (6)	2.71 (0.77-9.49)	2.06 (0.52-8.13)
ALL, 1–9.99 years (n=958)	0.9 (6)	0.4 (12)	1.53 (0.56-4.16)	1.02 (0.34-3.08)
ALL, 10–17.99 years (n=307)	2.0 (4)	0.8 (6)	2.00 (0.56-7.09)	1.56 (0.37-6.64)
AML (n=203)	3.9 (8)	3.3 (20)	2.25 (0.50-10.1)	1.44 (0.26-7.88)

OR: Odds ratio, 95% confidence interval

*Number of individuals diagnosed with autoimmune disease.

Supplementary table S3: Sensitivity analyses, odds ratios for acute leukemia by latency periods

	≤ 90d before	≤ 1y after	≤ 2.5y after	≤ 90d before / ≤ 1y after	≤ 90d before / ≤ 2.5y after	Special limitations[‡]
Any autoimmune disease, T1DM not included	OR (95% CI)	OR (95% CI)	OR (95% CI)	OR (95% CI)	OR (95% CI)	OR (95% CI)
Total (n=1563)	1.69 (1.29-2.23)	1.53 (1.15-2.04)	1.48 (1.10–1.98)	1.38 (1.03-1.86)	1.32 (0.97-1.79)	1.63 (1.24-2.15)
Sex						
Female (n=714)	1.69 (1.16-2.46)	1.60 (1.09-2.36)	1.58 (1.07-2.35)	1.50 (1.01-2.24)	1.48 (0.98-2.22)	1.66 (1.14-2.15)
Male (n=849)	1.70 (1.14-2.54)	1.46 (0.96-2.21)	1.36 (0.88-2.10)	1.25 (0.81-1.95)	1.14 (0.71-1.82)	1.59 (1.06-2.38)
Age						
0-0.99 years old (n=71)	13.14 (1.51-114)	16.12 (1.92-135)	15.00 (1.75-128)	13.14 (1.5-114)	12.00 (1.34-107)	13.14 (1.5-114)
1-9.99 years old (n=1081)	1.84 (1.29-2.62)	1.68 (1.17-2.42)	1.65 (1.14-2.39)	1.50 (1.02-2.19)	1.46 (0.98-2.16)	1.72 (1.20-2.46)
10-17.99 years old (n=410)	1.30 (0.82-2.06)	1.07 (0.65-1.75)	1.00 (0.60-1.68)	1.02 (0.61-1.69)	0.95 (0.56-1.61)	1.30 (0.82-2.06)
Type of leukemia						
ALL (n=1311)	1.80 (1.34-2.41)	1.57 (1.15-2.13)	1.48 (1.08-2.13)	1.45 (1.06-1.99)	1.36 (0.98-1.90)	1.73 (1.29-2.33)
ALL, female (n=597)	1.84 (1.23-2.74)	1.65 (1.09-2.59)	1.60 (1.09-2.50)	1.63 (1.07-2.48)	1.57 (1.02-2.42)	1.81 (1.22-2.69)
ALL, male (n=1081)	1.75 (1.13-2.71)	1.47 (0.93-2.34)	1.35 (0.83-2.20)	1.25 (0.77-2.04)	1.12 (0.66-1.88)	1.64 (1.05-2.56)
ALL, 0–0.99 years (n=46)	-	-	-	-	-	-
ALL, 1.5–5.99 years (n=710)	2.13 (1.36-3.33)	1.96 (1.24-3.11)	2.01 (1.27-3.19)	1.81 (1.12-2.91)	1.85 (1.15-2.91)	2.02 (1.29-3.17)
ALL, 1–9.99 years (n=958)	1.83 (1.26-2.64)	1.60 (1.09-2.35)	1.56 (1.05-2.32)	1.50 (1.01-2.23)	1.45 (0.96-2.19)	1.72 (1.18-2.50)
ALL, 10–17.99 years (n=307)	1.57 (0.94-2.60)	1.25 (0.72-2.18)	1.14 (0.64-2.03)	1.18 (0.68-2.08)	1.07 (0.60-1.93)	1.57 (0.94-2.60)
AML (n=203)	1.11e (0.46-2.63)	1.05 (0.44-2.48)	1.05 (0.44-2.48)	0.95 (0.38-2.37)	0.95 (0.38-2.37)	1.05 (0.44-2.48)

OR: Odds ratio, 95% confidence interval

[†] Excluded individuals with idiopathic thrombocytopenic purpura, Crohn’s disease or colitis ulcerosa diagnosis occurring less than 30 days apart from leukemia diagnosis or Addison’s disease or juvenile arthritis diagnosis occurring less than 60 days from leukemia diagnosis.

Supplementary table S4: Association between childhood acute leukemia and autoimmune diseases by composite groups

Composite group	Cases exposed *	Controls exposed*	Univariate model	Multivariate model
	% (n=)	% (n=)	OR* (95% CI)	OR* (95% CI)
IBD-disease[1]				
Total (n=1563)	1.2 (20)	1.1 (56)	1.26 (0.74-2.13)	1.47 (0.77-2.79)
Sex				
Female (n=714)	1.2 (9)	1.2 (26)	1.23 (0.56-2.7)	1.28 (0.47-3.49)
Male (n=849)	1.3 (11)	1.1 (30)	1.28 (0.63-2.6)	1.62 (0.7-3.73)
Age				
0-0.99 years old (n=71)	3.9 (3)	-	-	-
1-9.99 years old (n=1081)	1.0 (11)	0.9 (30)	1.28 (0.62-2.63)	1.17 (0.52-2.61)
10-17.99 years old (n=410)	1.4 (6)	2.1 (26)	0.82 (0.33-2.37)	1.94 (0.43-8.78)
Type of leukemia				
ALL (n=1311)	1.2 (16)	1.2 (50)	1.12 (0.63-2)	1.13 (0.55-2.33)
ALL, female (n=597)	1.3 (8)	1.2 (22)	1.27 (0.55-2.94)	1.13 (0.38-3.35)
ALL, male (n=714)	1.1 (8)	1.3 (28)	1 (0.45-2.24)	1.13 (0.43-2.97)
ALL, 0–0.99 years (n=46)	0	0	-	-
ALL, 1.5–5.99 years (n=710)	0.7 (5)	1.1 (25)	1.07 (0.38-3.04)	0.89 (0.28-2.84)
ALL, 1–9.99 years (n=958)	1.5 (15)	1.2 (35)	1.08 (0.5-2.36)	0.93 (0.38-2.26)
ALL, 10–17.99 years (n=307)	1.9 (6)	2.2 (21)	1 (0.4-2.52)	1.35 (0.33-5.46)
AML (n=203)	1.8 (4)	0.7 (5)	2.4 (0.64-8.94)	5.75 (1.05-31.66)
Rheumatic disease[2]				
Total (n=1563)	1.3 (21)	0.6 (27)	2.55 (1.34-4.87)	2.09 (0.92-4.74)
Sex				
Female (n=714)	1.5 (11)	0.7 (16)	2.25 (0.95-5.34)	2.52 (0.80-7.90)
Male (n=849)	1.1 (10)	0.4 (11)	3.00 (1.13-7.99)	1.73 (0.53-5.68)
Age				
0-0.99 years old (n=71)	2.6 (2)	0	-	-
1-9.99 years old (n=1081)	1.2 (14)	0.4 (14)	3.00 (1.30-6.92)	1.90 (0.70-5.19)
10-17.99 years old (n=410)	1.2 (5)	0.9 (11)	1.33 (0.41-4.33)	1.26 (0.22-7.17)
Type of leukemia				
ALL (n=1311)	1.3 (17)	0.6 (26)	2.21 (1.11-4.41)	1.81 (0.75-4.38)
ALL, female (n=597)	1.6 (10)	0.9 (16)	1.75 (0.69-4.44)	2.06 (0.62-6.83)
ALL, male (n=714)	1.0 (7)	0.5 (10)	3.00 (1.05-8.55)	1.55 (0.42-5.77)
ALL, 0–0.99 years (n=46)	0	0	-	-
ALL, 1.5–5.99 years (n=710)	1.1 (8)	0.2 (4)	2.25 (0.78-6.48)	1.93 (0.54-6.87)
ALL, 1–9.99 years (n=958)	1.2 (12)	0.2 (6)	2.18 (0.88-5.42)	1.35 (0.44-4.12)
ALL, 10–17.99 years (n=307)	0.6 (2)	0.5 (.5)	1.5 (0.45-4.98)	1.62 (0.26-10.09)

AML (n=203)	0.4 (1)	0.1 (1)	3 (0.19-48.0)	3.18 (0.2-51.19)
Thyroid disease[3]				
Total (n=1563)	7 (0.4)	12 (0.3)	1.75 (0.69-4.44)	2.29 (0.7-7.56)
Sex				
Female (n=714)	7 (0.9)	10 (0.4)	2.1 (0.8-5.52)	2.78 (0.8-9.65)
Male (n=849)	0	2 (0.1)	0	0
Age				
0-0.99 years old (n=71)	0	0	NA	NA
1-9.99 years old (n=1081)	5 (0.4)	7 (0.2)	2.14 (0.68-6.75)	3.4 (0.91-12.73)
10-17.99 years old (n=410)	2 (0.5)	5 (0.4)	1.2 (0.23-6.19)	0
Type of leukemia				
ALL (n=1311)	7 (0.5)	10 (0.2)	2.1 (0.8-5.52)	2.28 (0.69-7.53)
ALL, female (n=597)	7 (1.1)	8 (0.4)	2.63 (0.95-7.24)	2.74 (0.79-9.52)
ALL, male (n=714)	0	2 (0.1)	0	0
ALL, 0–0.99 years (n=46)	0	0	NA	NA
ALL, 1.5–5.99 years (n=710)	3 (0.4)	3 (0.1)	9.00 (0.94-86.5)	8.79 (0.9-85.37)
ALL, 1–9.99 years (n=958)	5 (0.5)	7 (0.2)	2.14 (0.68-6.75)	3.39 (0.91-12.71)
ALL, 10–17.99 years (n=307)	2 (0.6)	5 (0.5)	2.00 (0.33-11.97)	0
AML (n=203)	0	0	0	NA

1 Inflammatory bowel diseases: diagnosed with Crohn's disease or colitis ulcerosa

2 Diagnosed with juvenile idiopathic arthritis, rheumatoid arthritis or ankylosing spondylitis

3 Diagnosed with autoimmune thyroiditis or Basedow’s disease

OR: Odds ratio, 95% confidence interval

Supplementary table S5: Odds ratios for acute leukemia in individual autoimmune diseases

	Cases exposed* % (n=)	Controls exposed* % (n=)	Univariate model OR (95% CI)	Multivariate model OR (95% CI)
Colitis ulcerosa				
Total (n=1563)	0.9 (14)	0.7 (35)	1.20 (0.65-2.23)	1.28 (0.61-2.72)
Sex				
Female (n=714)	1.0 (7)	0.7 (14)	1.50 (0.61-3.72)	1.38 (0.47-4.04)
Male (n=849)	0.8 (7)	0.8 (21)	1.00 (0.43-2.35)	1.2 (0.42-3.44)
Age				
0-0.99 years old (n=71)	1 (1.4)	0	-	-
1-9.99 years old (n=1081)	9 (0.8)	19 (0.6)	1.42 (0.64-3.14)	1.19 (0.49-2.88)
10-17.99 years old (n=410)	4 (1.0)	16 (1.3)	0.75 (0.25-2.24)	1.02 (0.2-5.34)
Type of leukemia				
ALL (n=1311)	1.0 (13)	0.8 (32)	1.22 (0.64-2.32)	1.2 (0.55-2.63)
ALL, female (n=597)	1.1 (7)	0.6 (12)	1.75 (0.69-4.44)	1.52 (0.51-4.55)
ALL, male (n=714)	0.8 (6)	0.9 (20)	0.90 (0.36-2.24)	0.95 (0.31-2.98)
ALL, 0–0.99 years (n=46)	0	0	-	-
ALL, 1.5–5.99 years (n=710)	0.7 (5)	0.5 (12)	1.25 (0.44-3.55)	1.08 (0.34-3.42)
ALL, 1–9.99 years (n=958)	0.8 (8)	0.6 (18)	1.33 (0.58-3.07)	1.08 (0.42-2.78)
ALL, 10–17.99 years (n=307)	1.3 (4)	1.5 (14)	0.86 (0.28-2.6)	1.07 (0.2-5.57)
AML (n=203)	0.4 (1)	0.3 (2)	1.00 (0.1-9.61)	2.96 (0.19-47.45)
Celiac disease				
Total (n=1563)	0.6 (9)	0.6 (28)	0.96 (0.46-2.04)	0.64 (0.24-1.68)
Sex				
Female (n=714)	0.8 (6)	0.7 (15)	1.2 (0.47-3.09)	0.72 (0.2-2.54)
Male (n=849)	0.4 (3)	0.5 (13)	0.69 (0.2-2.43)	0.55 (0.12-2.48)
Age				
0-0.99 years old (n=71)	1.4 (1)	0	-	-
1-9.99 years old (n=1081)	0.6 (7)	0.6 (21)	1 (0.43-2.35)	0.59 (0.2-1.72)
10-17.99 years old (n=410)	0.2 (1)	0.6 (7)	0.43 (0.05-3.48)	0
Type of leukemia				
ALL (n=1311)	0.6 (8)	0.6 (22)	1.09 (0.49-2.45)	0.76 (0.28-2.05)
ALL, female (n=597)	1.0 (6)	0.7 (13)	1.38 (0.53-3.64)	0.78 (0.22-2.8)
ALL, male (n=714)	0.3 (2)	0.4 (9)	0.67 (0.14-3.09)	0.75 (0.16-3.53)
ALL, 0–0.99 years (n=46)	2.2 (1)	0	-	-

ALL, 1.5–5.99 years (n=710)	0.7 (5)	0.6 (13)	1.15 (0.41-3.24)	1.02 (0.32-3.21)
ALL, 1–9.99 years (n=958)	0.7 (7)	0.6 (17)	1.24 (0.51-2.98)	0.69 (0.23-2.04)
ALL, 10–17.99 years (n=307)	0	0.5 (5)	0	0
AML (n=203)	0.5 (1)	0.8 (5)	0.6 (0.07-5.14)	0
Psoriasis				
Total (n=1563)	0.6 (10)	0.6 (30)	1 (0.49-2.05)	0.97 (0.31-3.02)
Sex				
Female (n=714)	0.8 (6)	0.6 (13)	1.38 (0.53-3.64)	2.14 (0.48-9.59)
Male (n=849)	0.5 (4)	0.7 (17)	0.71 (0.24-2.1)	0.39 (0.05-3.63)
Age				
0-0.99 years old (n=71)	1.4 (1)	0.5 (1)	3 (0.19-47.96)	-
1-9.99 years old (n=1081)	0.4 (4)	0.4 (13)	0.92 (0.3-2.83)	0.57 (0.13-2.62)
10-17.99 years old (n=410)	1.2 (5)	1.3 (16)	0.94 (0.34-2.56)	1.56 (0.14-17.2)
Type of leukemia				
ALL (n=1311)	0.6 (8)	0.6 (22)	1.09 (0.49-2.45)	1.28 (0.39-4.17)
ALL, female (n=597)	1.0 (6)	0.6 (11)	1.64 (0.61-4.42)	2.1 (0-47-9.44)
ALL, male (n=714)	0.3 (2)	0.5 (11)	0.55 (0.12-2.46)	0.62 (0.07-5.32)
ALL, 0–0.99 years (n=46)	2.2 (1)	0	-	-
ALL, 1.5–5.99 years (n=710)	0.4 (3)	0.3 (7)	1.29 (0.33-4.97)	0.64 (0.14-2.96)
ALL, 1–9.99 years (n=958)	0.4 (4)	0.4 (12)	1 (0.32-3.1)	1.14 (0.22-5.91)
ALL, 10–17.99 years (n=307)	1.0 (3)	1.1 (10)	0.9 (0.25-3.27)	
AML (n=203)	0.5 (1)	0.8 (5)	0.6 (0.07-5.14)	0
Crohns disease				
Total (n=1563)	0.4 (6)	0.4 (21)	0.86 (0.35-2.12)	1.39 (0.47-4.09)
Sex				
Female (n=714)	0.3 (2)	0.5 (12)	0.5 (0.11-2.23)	0.56 (0.06-4.79)
Male (n=849)	0.5 (4)	0.3 (9)	1.33 (0.41-4.33)	2.2 (0.58-8.27)
Age				
0-0.99 years old (n=71)	2.6 (2)	0	-	-
1-9.99 years old (n=1081)	0.2 (2)	0.3 (11)	0.55 (0.12-2.46)	0.69 (0.15-3.24)
10-17.99 years old (n=410)	0.5 (2)	0.8 (10)	0.6 (0.13-2.74)	1.49 (0.13-16.7)
Type of leukemia				
ALL (n=1311)	0.2 (3)	0.4 (18)	0.5 (0.15-1.7)	0.61 (0.13-2.82)
ALL, female (n=597)	0.2 (1)	0.5 (10)	0.3 (0.04-2.34)	0
ALL, male (n=714)	0.3 (2)	0.4 (8)	0.75 (0.16-3.53)	1.35 (0.25-7.44)
ALL, 0–0.99 years	0	0	NA	NA

(n=46)				
ALL, 1.5–5.99 years (n=710)	0	0,6 (13)	0	0
ALL, 1–9.99 years (n=958)	0.7 (7)	0.6 (17)	0.27 (0.04-2.11)	0.34 (0.04-2.72)
ALL, 10–17.99 years (n=307)	0.6 (2)	0.7 (7)	0.86 (0.18-4.13)	2.77 (0.17-44.88)
AML (n=203)	1.3 (3)	0.4 (3)	3 (0.61-14.86)	8.52 (0.2-51.19)
Addison's disease				
Total (n=1563)	0.5 (8)	0.0 (1)	24 (3-191.86)	20.92 (2.57-170.34)
Sex				
Female (n=714)	0.4 (3)	0	-	-
Male (n=849)	0.6 (5)	0.0 (1)	15 (1.75-128.39)	11.46 (1.27-103.25)
Age				
0-0.99 years old (n=71)	1.4 (1)	0		
1-9.99 years old (n=1081)	0.5 (5)	0.0 (1)	15 (1.75-128.39)	15.79 (1.84-135.32)
10-17.99 years old (n=410)	0.2 (2)	0		
Type of leukemia				
ALL (n=1311)	0.6 (8)	0.0 (1)	24 (3-191.89)	20.92 (2.57-170.36)
ALL, female (n=597)	0.5 (3)	0		
ALL, male (n=714)	0.7 (5)	0.0 (1)	15 (1.75-128.39)	11.46 (1.27-103.17)
ALL, 0–0.99 years (n=46)	2.2 (1)	0		
ALL, 1.5–5.99 years (n=710)	0.6 (4)	0.0 (1)	12 (1.34-107.36)	12.2 (1.36-109.3)
ALL, 1–9.99 years (n=958)	0.5 (5)	0.0 (1)	15 (1.75-128.39)	15.95 (1.86-136.76)
ALL, 10–17.99 years (n=307)	0.7 (2)	0.0 (0)		
AML (n=203)	0	0	NA	NA
Juvenile arthritis				
Total (n=1563)	0.8 (12)	0.2 (9)	4 (1.69-9.49)	3.46 (1.37-8.76)
Sex				
Female (n=714)	0.7 (7)	0.2 (9)	4.2 (1.33-13.23)	3.7 (1.03-13.28)
Male (n=849)	0.6 (5)	0.2 (4)	3.75 (1.01-13.96)	3.25 (0.83-12.65)
Age				
0-0.99 years old (n=71)	2.6 (2)	0	-	-
1-9.99 years old (n=1081)	0.7 (8)	0.1 (4)	3.43 (1.24-9.45)	2.79 (0.92-8.48)
10-17.99 years old (n=410)	0.5 (2)	0.2 (3)	3 (0.42-21.3)	2.75 (0.37-20.6)
Type of leukemia				
ALL (n=1311)	0.8 (11)	0.2 (8)	3.37 (1.3-8.75)	3.16 (1.15-8.66)
ALL, female (n=597)	1.0 (6)	0.3 (5)	3 (0.87-10.36)	3.16 (1.15-8.66)
ALL, male (n=714)	0.7 (5)	0.1 (3)	4 (0.9-17.87)	3.03 (0.8-11.44)

ALL, 0–0.99 years (n=46)	4.3 (2)	0		
ALL, 1.5–5.99 years (n=710)	0.7 (5)	0.1 (3)	2.4 (0.64-8.94)	2.97 (0.74-11.96)
ALL, 1–9.99 years (n=958)	0.9 (9)	0.1 (4)	2.14 (0.68-6.75)	1.98 (0.59-6.69)
ALL, 10–17.99 years (n=307)	0.6 (2)	0.2 (2)	6 (0.54-66.17)	4.9 (0.42-56.79)
AML (n=203)	0.4 (1)	0.1 (1)	3 (0.19-47.96)	3.18 (0.2-51.19)
Idiopathic trombosytopenic purpura				
Total (n=1563)	0.6 (10)	0.1 (7)	4.72 (1.71-13.08)	8.36 (2.25-31.06)
Sex				
Female (n=714)	0.7 (5)	0.3 (6)	2.71 (0.77-9.49)	3.47 (.0.77-15.67)
Male (n=849)	0.6 (5)	0.0 (1)	15 (1.75-128.39)	
Age				
0-0.99 years old (n=71)	0	0	NA	NA
1-9.99 years old (n=1081)	0.6 (7)	0.1 (4)	6.42 (1.64-25.09)	9.36 (1.93-45.44)
10-17.99 years old (n=410)	0.7 (3)	0.2 (3)	3 (0.61-14.86)	6.5 (0.59-27.43)
Type of leukemia				
ALL (n=1311)	0.6 (8)	0.2 (6)	4.47 (1.45-13.78)	6.41 (1.64-25)
ALL, female (n=597)	0.5 (3)	0.3 (5)	1.92 (0.42-8.82)	1.62 (0.26-9.9)
ALL, male (n=714)	0.7 (5)	0.0 (1)	15 (1.75-128.39)	
ALL, 0–0.99 years (n=46)			NA	NA
ALL, 1.5–5.99 years (n=710)	0.7 (5)	0.2 (4)	4.46 (1.05-18.95)	6.49 (1.24-33.89)
ALL, 1–9.99 years (n=958)	0.5 (5)	0.1 (4)	4.46 (1.05-18.95)	6.39 (1.22-33.44)
ALL, 10–17.99 years (n=307)	1.0 (3)	0.2 (2)	4.5 (0.75-26.93)	6.42 (0.58-71.12)
AML (n=203)	0.5 (1)	0.2 (1)	3 (0.19-47.96)	
Ankylosing spondylitis				
Total (n=1563)	0.1 (2)	0.2 (9)	0.67 (0.14-3.09)	-
Sex				
Female (n=714)	0.1 (1)	0.2 (4)	0.75 (0.16-3.53)	-
Male (n=849)	0.1 (1)	0.2 (5)	0.6 (0.07-5.14)	-
Age				
0-0.99 years old (n=71)	0	0	NA	NA
1-9.99 years old (n=1081)	0.1 (1)	0.1 (4)	0.75 (0.08-6.71)	-
10-17.99 years old (n=410)	0.2 (1)	0.4 (5)	0.6 (0.07-5.14)	-
Type of leukemia				
ALL (n=1311)	0.1 (2)	0.2 (9)	0.67 (0.14-3.09)	-
ALL, female (n=597)	0.2 (1)	0.2 (4)	0.75 (0.08-6.71)	-

ALL, male (n=714)	0.1 (1)	0.2 (5)	0.6 (0.07-5.14)	-
ALL, 0–0.99 years (n=46)	0	0	NA	NA
ALL, 1.5–5.99 years (n=710)	0	0	NA	NA
ALL, 1–9.99 years (n=958)	0	0.1 (2)	0.75 (0.08-6.71)	-
ALL, 10–17.99 years (n=307)	0.6 (2)	0.3 (3)	0.75 (0.07-5.14)	-
AML (n=203)	0	0	NA	NA
Rheumatoid arthritis				
Total (n=1563)	0.4 (7)	0.2 (9)	2.33 (0.87-6.27)	1.22 (0.30-4.97)
Sex				
Female (n=714)	0.4 (3)	0.3 (7)	1.29 (0.33-4.97)	0.51 (0.05-4.67)
Male (n=849)	0.5 (4)	0.1 (2)	6.00 (1.10-32.76)	3.00 (0.42-21.66)
Age				
0-0.99 years old (n=71)	0	0	NA	NA
1-9.99 years old (n=1081)	0.4 (5)	0.2 (6)	2.50 (0.76-8.19	1.06 (0.20-5.48)
10-17.99 years old (n=410)	0.5 (2)	0.2 (3)	2.00 (0.33-11.97)	2.15 (0.12-37.49)
Type of leukemia				
ALL (n=1311)	0.3 (4)	0.2 (9)	1.67 (0.56-4.97)	0.83 (0.16-4.2)
ALL, female (n=597)	0.5 (3)	0.4 (7)	0.86 (0.18-4.13)	0.51 (0.06-4.72)
ALL, male (n=714)	0.1 (1)	0.1 (2)	4.50 (0.75-26.93)	1.7 (0.15-18.83)
ALL, 0–0.99 years (n=46)	0	0	NA	NA
ALL, 1.5–5.99 years (n=710)	0.4 (3)	0.0 (1)	2.25 (0.50-10.05)	0.94 (0.1-9.09)
ALL, 1–9.99 years (n=958)	0.3 (3)	0	1.5 (0.38-6.00)	0.56 (0.06-4.80)
ALL, 10–17.99 years (n=307)	0.3 (1)	0	2 (0.33-11.97)	2.09 (0.12-36.62)
AML (n=203)	0	0	NA	NA

OR: Odds ratio, 95% confidence interval

*Number of individuals diagnosed with autoimmune disease.

Supplementary table S6: Pooled analyses including type 1 diabetes mellitus

	Cases exposed*	Controls exposed*	Univariate model	Multivariate model
	% (n=)	% (n=)	OR (95% CI)	OR (95% CI)
Any autoimmune disease				
Total (n=1533)	7.0 (110)	3.9 (185)	1.80 (1.40-2.30)	1.88 (1.40-2.52)
Sex				
Female (n=700)	8.0 (57	4.6 (98)	1.80 (1.27-2.54)	1.80 (1.18-2.75)
Male (n=833)	6.2 (53)	3.4 (87)	1.79 (1.26-2.56)	1.98 (1.31-2.97)
Age				
0-0.99 years old (n=71)	9.9 (7)	1.9 (4)	6.17 (1.58-24.15)	8.07 (1.60-40.59)
1-9.99 years old (n=1059)	6.3 (68)	3.5 (113)	1.84 (1.34-2.53)	1.75 (1.24-2.46)
10-17.99 years old (n=402)	8.5 (35)	5.5 (68)	1.50 (0.98-2.30)	1.76 (0.93-3.34)
Type of leukemia				
ALL (n=1282)	7.2 (95)	4.0 (158)	1.84 (1.41-2.41)	1.82 (1.33-2.49)
ALL, female (n=583)	8.7 (52)	4.9 (87)	1.86 (1.29-2.69)	1.69 (1.08-2.62)
ALL, male (n=699)	6.0 (43)	3.3 (71)	1.82 (1.23-2.70)	1.99 (1.27-3.13)
ALL, 0–0.99 years (n=46)	2.2 (1)	2.2 (3)	4.82 (0.86-26.96)	4.69 (0.83-26.54)
ALL, 1.5–5.99 years (n=693)	6.3 (45)	3.1 (66)	2.11 (1.41-3.16)	2.01 (1.31-3.08)
ALL, 1–9.99 years (n=936)	6.2 (59)	3.6 (104)	1.72 (1.22-2.41)	1.62 (1.12-2.33)
ALL, 10–17.99 years (n=300)	10.4 (132)	5.5 (51)	1.92 (1.21-3.05)	2.35 (1.19-4.64)
AML (n=202)	4.9 (10)	3.8 (23)	1.17 (0.49-2.79)	2.01 (0.8-5.08)

OR: Odds ratio, 95% confidence interval

*Number of individuals diagnosed with autoimmune disease.

Supplementary figures

Supplementary figure S1: Timing of autoimmune disease diagnoses relative to leukemia.

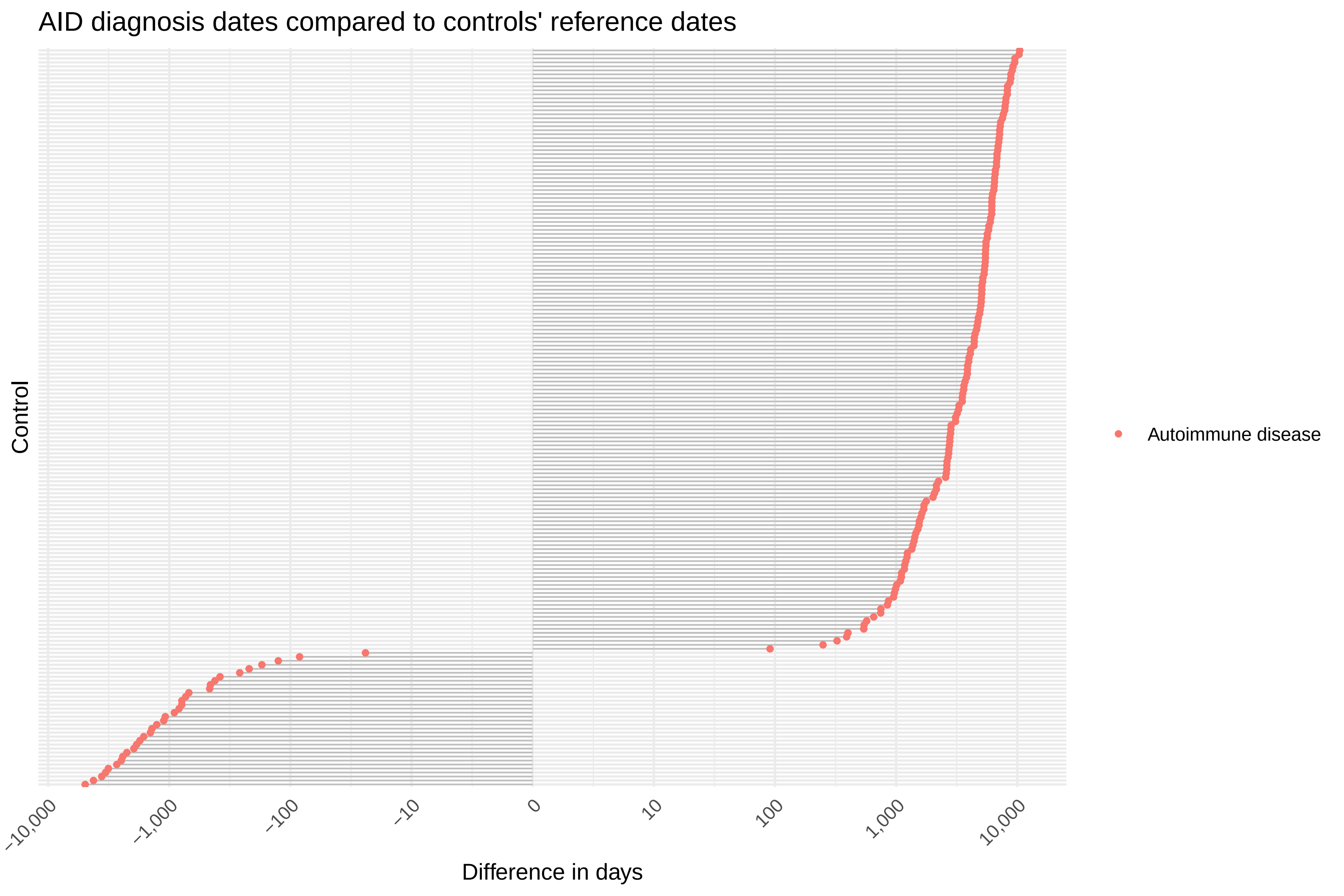
AID diagnosis dates compared to controls' reference dates
Control
Difference in days
−10,000
−1,000
−100
−10
0
10
100
1,000
10,000
Autoimmune disease

Supplementary figure S2: Timing of autoimmune disease diagnoses in controls relative to reference date.

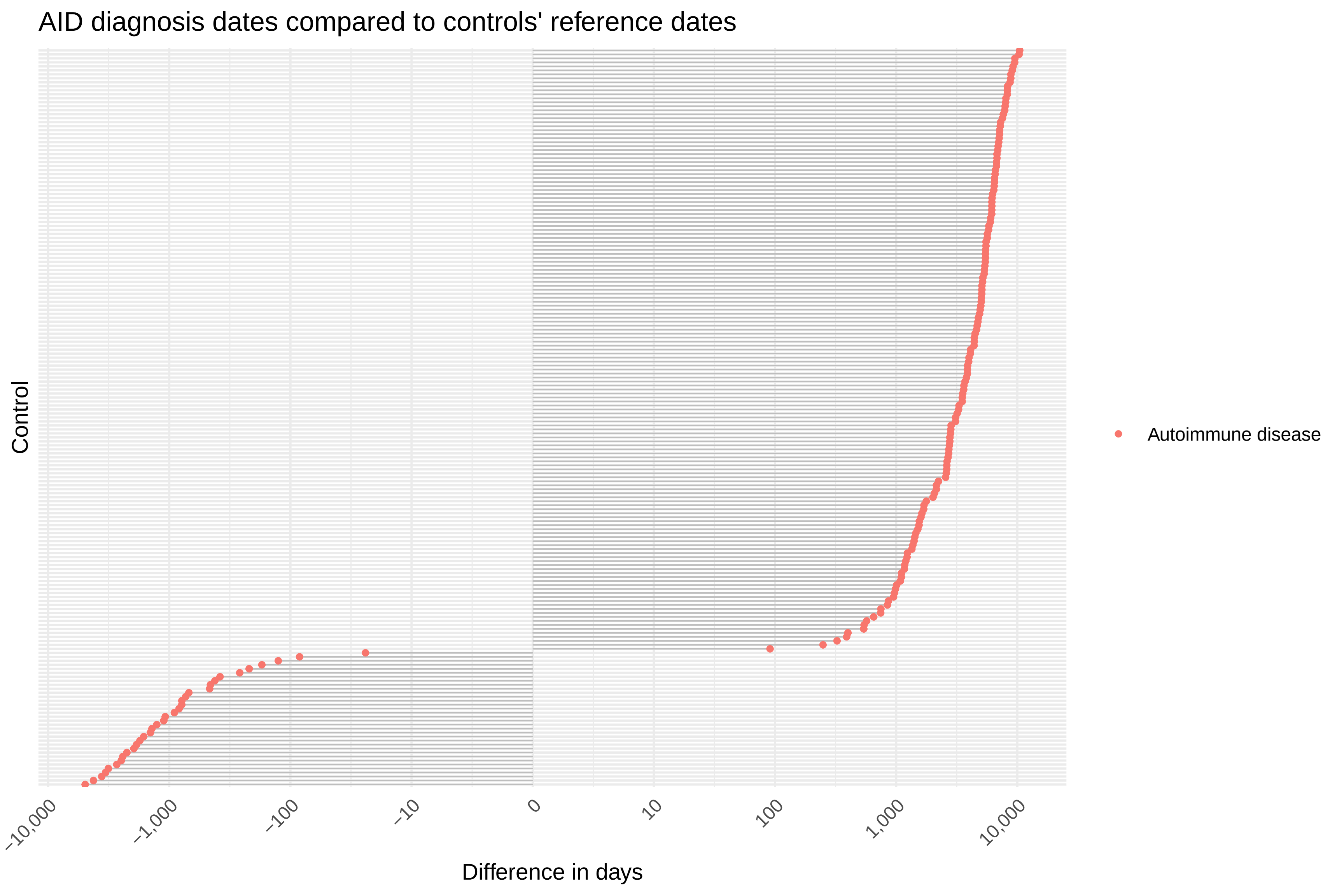
AID diagnosis dates compared to controls' reference dates
Control
Difference in days
−10,000
−1,000
−100
−10
0
10
100
1,000
10,000
Autoimmune disease
